# Safety of outpatient anterior cervical discectomy and fusion: a systematic review and meta-analysis

**DOI:** 10.1186/s40001-016-0229-6

**Published:** 2016-08-31

**Authors:** Dexiang Ban, Yang Liu, Taiwei Cao, Shiqing Feng

**Affiliations:** Department of Orthopedics, Tianjin Medical University General Hospital, 154 Anshan Road, Heping District, Tianjin, 300052 People’s Republic of China

**Keywords:** ACDF, Outpatient, Inpatient, Complication

## Abstract

**Background:**

Anterior cervical discectomy and fusion (ACDF) is one of the most prevalent spine surgeries and neurosurgical procedures performed to treat a variety of disorders in the cervical spine. Over the last several years, ACDF has been done in the outpatient setting for less invasive approaches and exposures, as well as modified anesthetic and pain management techniques. Despite the fact that it may be innocuous in other parts of the body, complications in the spine can literally be fatal. The objective of this article is to evaluate the safety of outpatient surgery compared with inpatient surgery in the cervical spine for adult patients.

**Methods:**

The multiple databases including Pubmed, Springer, EMBASE, EBSCO and China Journal Full-text Database were adopted to search for the relevant studies in English or Chinese. Full-text articles involving to the safety of outpatient cervical spine surgery were selected. Review Manager 5.0 was adopted to estimate the effects of the results among selected articles. Forest plots, sensitivity analysis and bias analysis for the articles included were also conducted. Chi-square tests were conducted with SPSS 20.0 software.

**Results:**

Finally, 12 articles were included. The results of meta-analysis suggested that in the articles included, no death occurred, and compared with inpatient surgery, outpatient surgery has a similar risk (RR = 0.99, 95 % CI [0.98, 1.00], *P* = 0.02; *P* for heterogeneity = 0.47, *I*^2^ = 0 %). An *I*^2^ value of 0 % indicates no heterogeneity observed. All complications were occurred in both outpatients and inpatients. Among the studies selected, after the outpatient spine surgery, the highest incidences of complication were dysphagia (18/29) and hematoma (4/29). Compared with the overall complication rate in inpatient group, no significant difference was observed (*x*^2^ = 1.820, *P* = 0.177).

**Conclusion:**

In this study, outpatient surgery has a similar risk with inpatient surgery, and no difference of morbidity between outpatient and inpatient was found. Because of short operative time and moderate postoperative pain, we believe that outpatient cervical spine surgery is a safe and convenient alternative procedure, which also decrease the cost of care. Besides, postoperative complications including dysphagia and hematoma should be noticed.

## Background

The number of surgeries performed for degenerative cervical spine disease each year continues to increase, and an overall increase in cervical spine surgery in recent years have been observed [1, 2]. Anterior cervical discectomy and fusion (ACDF), one of the most prevalent spine surgeries and neurosurgical procedures, is performed to treat a variety of disorders in the cervical spine [3, 4]. ACDS is widely used to treat nerve root or spinal cord compression by decompressing the spinal cord and nerve roots of the cervical spine with a discectomy to stabilize the corresponding vertebrae. Since originally described by Smith [5] in 1958, ACDF has been considered as the gold standard for many degenerative cervical spine diseases owing to its relative simplicity, minimal risk, and reliability [6]. As the aging population is growing, the frequency of its performance increases rapidly.

Traditionally, spine surgery in inpatient setting is well established [7–10]. But with the increasing medical costs and the mounting number of surgery procedures, outpatient surgeries have become increasingly important [11], and some studies [12–14] suggested that ACDF may be well-suited to be performed in the outpatient setting, concerning trends in spinal surgery towards less invasive approaches and exposures, as well as modified anesthetic and pain management techniques.

Existing studies have proven that there is no compromise in safety and efficacy of anterior cervical discectomy and fusion when performed as an outpatient [14, 15]. Complications occurred in these studies were not due to the outpatient setting or influenced in any way by the outpatient setting. But the application of outpatient ACDF remains controversial, which is mainly about the incidence of complications. Despite the fact that it may be innocuous in other parts of the body, complications in the spine can literally be fatal. After cervical spine surgery, a common complication like hematoma may compromise the airway and result in hypoxic complications if not addressed rapidly. The potential problems make ambulatory surgery with an anterior approach potentially problematic. For this reason most spine surgeons hesitated to do the procedure on an outpatient basis initially.

The objective of this article is to evaluate the safety of outpatient surgery compared with inpatient surgery in the cervical spine for adult patients. To compare the incidence of complications between outpatient surgery and inpatient surgery, it is necessary to perform a comprehensive literature search and meta-analysis. Further investigations about the safety and complication rates of ambulatory surgery will undoubtedly increase surgeon confidence of both medical staffs and patients.

## Methods

### Search strategy

Related citations about the outpatient cervical spine surgery were systematically searched and a systematic review of the literature was undertaken for articles published through February 2016 among multiple electronic databases including Pubmed, Springer, EMBASE, EBSCO and China Journal Full-text Database. The comparative studies evaluating the safety of inpatient versus outpatient surgery in the cervical spine were selected in all publication status including published, unpublished, in press and in progress. Two authors (Yang Liu and Taiwei Cao) in our team searched the literature independently. The following keywords were used in our search work: (1) outpatient OR ambulatory; (2) anterior cervical discectomy and fusion OR ACDF OR cervical spine surgery; (3) complication. All these keywords were assembled with the Boolean operator “and” to search for the articles related in the multiple electronic databases. All the citations searched out were screened for the further selection.

### Citation selection

Another two researchers selected the citations in this process, independently and attentively. All the primary searched results (full text or abstract) were screened to identify the corresponding studies that may require further retrieval. These relevant studies included in this study must meet the following inclusion criteria: (1) the study must be a randomized control trials study; (2) outpatient spine surgery conduct; (3) complications involved; (4) outpatient spine surgery versus inpatient spine surgery; (5) patients with 18 years of age or older; (6) availability of full text.

Two authors (Dexiang Ban and Yang Liu) scanned the titles and abstracts of the identified articles to check whether the study was likely to be relevant. Studies that were considered to be included in the study were obtained as full text articles and independently assessed for inclusion by the same two authors. After the primary selection, these two researches met and reviewed their selections for agreement. If any difference existed, a third person (Shiqing Feng) was involved to discussion. At last, 12 relevant original articles were selected in this study.

### Data extraction

Coding sheets in Microsoft Excel 2010 were developed before data extracting. Another two reviewers independently read the full text of the articles and extracted the characteristics from each study. In the first part, the meta-analysis about complications of outpatient surgery and inpatient surgery was conducted, and the first author’s name, year of publication, year of onset, follow-up time, events without complications, total sample size and matched factors of related articles were collected. In the second part, the amount and type of complications in different citations was summarized. If any problems of poor agreement occurred, these two reviewers solved after a discussion with a third investigator.

### Statistical analysis

Review Manager 5.0 (The Cochrane Collaboration, 2011) was adopted to estimate the effects of outpatient spine surgery among selected articles. Related risk (RR) with 95 % confidence intervals (CIs) was calculated. Heterogeneity was investigated with the heterogeneity *I*^2^ statistic in this study. The value of *I*^2^ statistic reflects the levels of heterogeneity. In general, *I*^2^ value at 25, 50 and 75 % were considered as the boundary value of low, moderate and high amounts of heterogeneity, respectively. When the moderate or high heterogeneity was obtained, which means the heterogeneity *I*^2^ statistic >50 %, a random-effect model was adopted, otherwise a fixed-effect model was chose. The *P* values reported in this meta-analysis are carried out from the *x*^2^ test. All these *P* values are two-sided and *P* < 0.05 was considered to represent statistically significant. In addition, sensitivity analysis and bias analysis of the articles were conducted to examine the quality of articles and the influence on the meta-analysis. To estimate possible publication bias, a funnel plot was used.

A comparison of the complication rates between the outpatient group and meta-analysis derived comparison group was carried out using *x*^2^ test with SPSS 20.0 software (IBM Corporation, 2013). Statistically significance was defined at *P* < 0.05.

## Results

### Search results

**Fig. 1:**
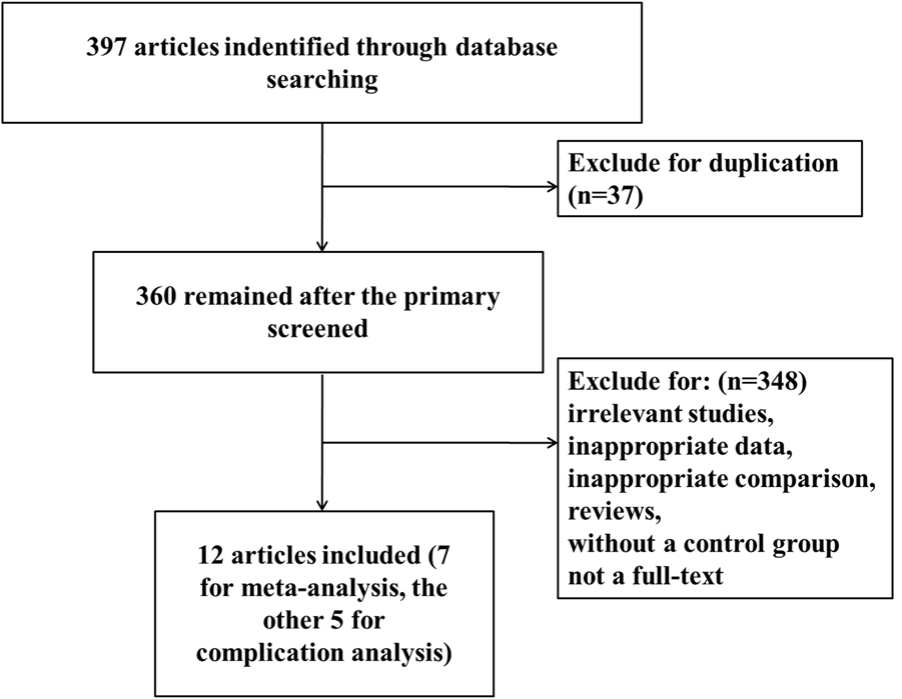
Flow diagram of the study selection which shows the number of citations identified, excluded and included in final analysis

A total of 397 articles were initially located in these electronic databases after the primary selection. Twelve [16–27] of those met all of the inclusion criteria. The other 388 articles were excluded for duplication, irrelevant studies, inappropriate data, inappropriate comparison, reviews, without a control group or not a full-text. Among these nine included articles, seven involved in the comparison of the complication rate between outpatient surgery setting and inpatient surgery setting. Figure 1 shows the flow diagram that reflects the search process. Among the 12 article, seven [16, 18, 21, 22, 24, 25, 27] were subsumed into the meta-analysis, and the other five [17, 19, 20, 23, 26] were included to present the complications of the outpatient surgery.

### Characteristics of included studies

In the meta-analysis, a total of 3435 patients were included in the seven studies. Thousand seven hundred and two were treated in outpatient surgery setting, while the other 1733 were in inpatient surgery setting. Detailed characteristics of the included studies were provided in Table 1. The first author’s name, year of publication, year of onset, follow-up time, events without complications, total sample size and matched factors were presented in the table. All these articles were published from 1996 to 2016. The sample size ranges from 86 to 1442. All patients in these studies were adults.

**Table 1:** Characteristic of the included studies

Author	Year	Year of onset	Follow-up time	Inpatient		Outpatient		Matched factors
				Events	Total	Events	Total	
Adamson [16]	2014	2006–2013	90 days	270	274	621	629	ACDF level and other disease
Liu [18]	2009	August 2005–May 2007	62.4 days (mean)	60	64	45	45	ACDF level and operation condition
McGirt [27]	2015	2005–2011	30 days	630	650	781	792	Age, other disease, operation condition and ASA grades
Silvers [21]	1996	–	1.3 (outpatient) and 1.6 (inpatient) years	52	53	49	50	None
Stieber [22]	2005	1998–2002	21 days	49	56	27	30	Age
Trahan [24]	2011	November 2005–April 2009	6 h	58	58	58	59	Age
Walid [25]	2010	–	–	562	578	96	97	Age and other disease

### Risk of bias

The risk of bias table in this meta-analysis was present in Table 2. Because of the particularity of the operation, the places where the operations of outpatient setting and inpatient setting were conducted were different, so we thought that high risk of blinding of participants and personnel was existed. While the risks of incomplete outcome data and selective reporting were relatively low.

**Table 2:** The risk of bias table in this meta-analysis

	Adamson [16]	Liu [18]	McGirt [27]	Silvers [21]	Stieber [22]	Trahan [24]	Wohns [25]
Random sequence generation	Not	Low	Low	Low	Low	High	Low
Allocation concealment	Low	Low	Low	Low	Not	Low	High
Blinding of participants and personnel	High	High	High	High	High	High	High
Blinding of outcome assessment	Low	High	Low	High	Low	Low	Low
Incomplete outcome data	Low	Low	Not	Low	Low	Low	Low
Selective reporting	Low	Low	Low	Low	Low	Not	Low
Other bias	Not	Low	Not	Not	Not	Low	Low

### Results of meta-analysis

**Fig. 2:**
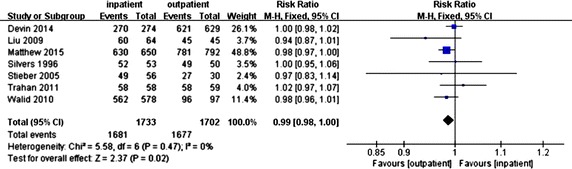
Forest plots for complication in outpatient surgery and inpatient surgery

Forest plots for complications in outpatient surgery and inpatient surgery were shown in Fig. 2. The results of meta-analysis suggested that in the articles included, compared with inpatient surgery, outpatient surgery has a similar risk (RR = 0.99, 95 % CI [0.98, 1.00], *P* = 0.02; *P* for heterogeneity = 0.47, *I*^2^ = 0 %). And all the seven articles included in the meta-analysis have a similar result. According to the results above, no heterogeneities of complications in outpatient and inpatient surgery was observed (*I*^2^ = 0 %), and the fixed-effect model was used.

### Results of publication bias

**Fig. 3:**
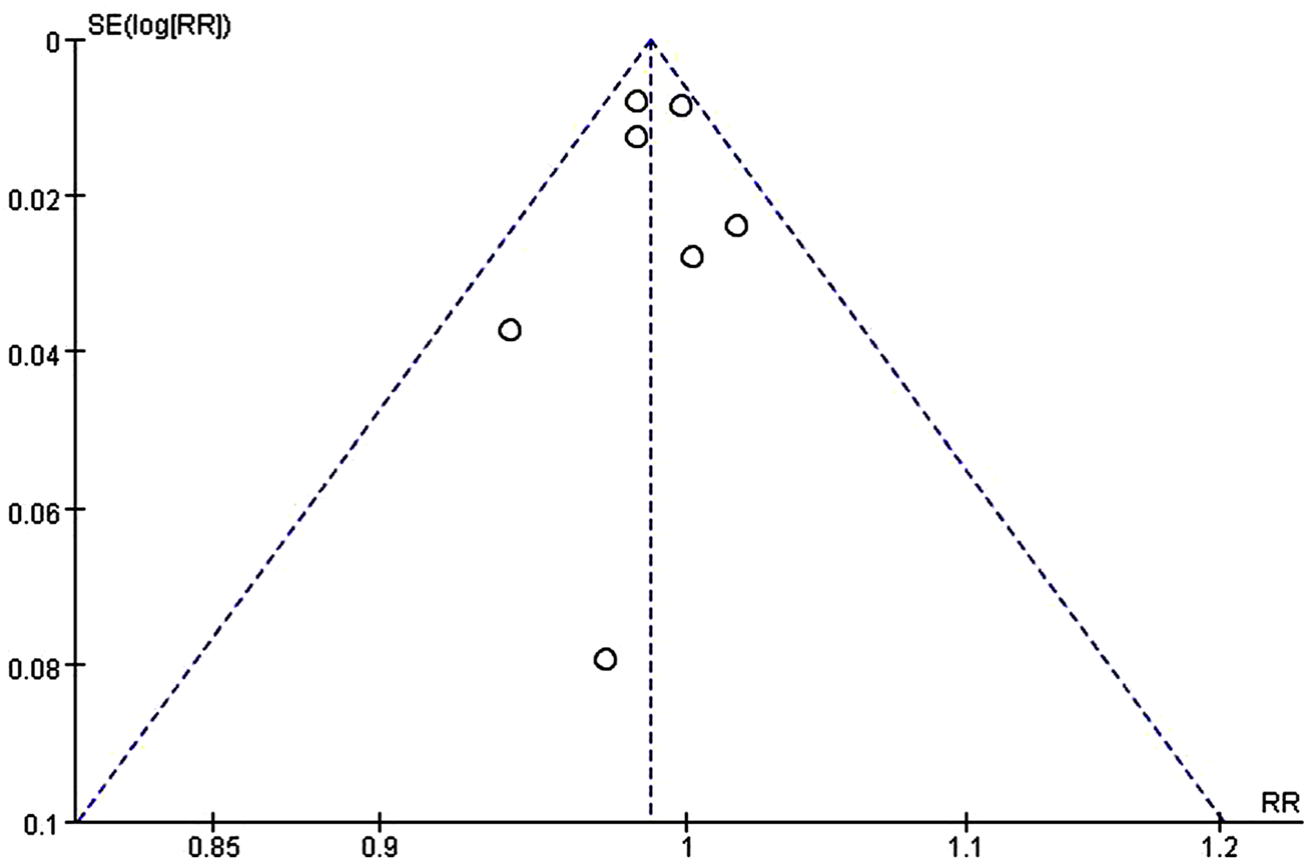
A funnel plot for the articles included

**Fig. 4:**
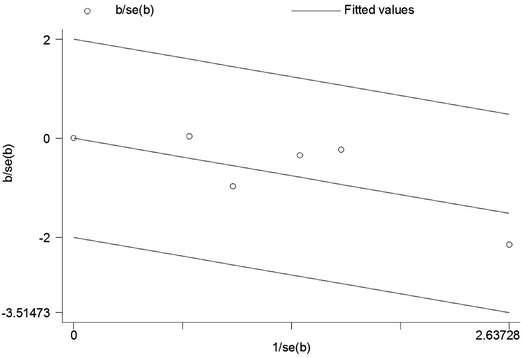
A Galbraith radial plot for the articles included

Both funnel plot and Galbraith radial plot for the articles included were performed (Figs. 3, 4). These two figures have shown that all the articles included were in the confidence limit. And also the Egger’s test presented in Table 3 showed that no publication bias was observed (*P* = 0.785).

**Table 3:** Egger’s test of the publication bias

Std_eff	Coef.	Std. err.	*t*	*P* > |*t*|	[95 % conf. interval]
Slope	5.844155	1.1449	5.10	0.015	(2.200572, 9.487739)
Bias	−0.0157073	0.0527741	−0.30	0.785	(−0.1836579, 0.1522433)

### Complication of the outpatient surgery

In all 12 articles included, the characteristics of complications in outpatient surgery were presented in Table 4. No death was observed in both outpatient and inpatient surgery, and no complication were found in the studies of Liu, Shin, Tally and Wohns. The overall complication rate was 1.71 % (29/1693). Dysphagia (18/29) was the most common complication, followed by hematoma (4/29), swelling (2/29), infection (2/29) and pain (2/29). Other complications such as nausea, cervical swelling, vocal paralysis were observed on each case.

**Table 4:** The characteristics of complications in outpatient surgery

Author	Total	Events with complication	Complication rate (%)	Complication
Adamson [16]	629	13	2.07	Dysphagia (*n* = 11), surgical site infection (*n* = 1), hematoma (*n* = 1)
Lied [17]	96	4	4.17	Hematoma (*n* = 1), dysphagia (*n* = 2), deterioration of neurological function (*n* = 1)
Liu [18]	45	0	0.00	None
Sheperd [19]	152	6	3.95	Pain (*n* = 2), dysphagia (*n* = 2), nausea (*n* = 1), cervical swelling (*n* = 1)
Shin [20]	390	0	0.00	None
Silvers [21]	50	1	2.00	Vocal paralysis (*n* = 1)
Stiber [22]	30	3	10.00	Dysphagia (*n* = 3)
Tally [23]	119	0	0.00	None
Trahan [24]	59	1	1.69	Neck swelling and difficulty breathing (*n* = 1)
Walid [25]	97	1	1.03	Infection (*n* = 1)
Wohns [26]	26	0	0.00	None

To compare the incidence of complications between outpatient and inpatient, Villavicencio [28] conducted a comprehensive literature search and meta-analysis. 633 patients of nine studies were selected in the analysis and six overall complications were identified in the comparison group. The complication rate was 0.95 %. The difference of complication rate between the outpatient and meta-analysis derived comparison group was compared by Chi-square test, and the results showed that no significant differences were observed, which suggested that the complication rate of surgery on outpatient basis was not higher than that of surgery on inpatient basis (*x*^2^ = 1.820, *P* = 0.177).

## Discussion

In 1987, outpatient spine surgery was first reported for the lumbar spine, and in 1996 reported for anterior cervical spine surgery. However, the numbers of patients treated on an outpatient basis were small for the suspect of safety [29]. Due to the optimization of facilities and systems for outpatient surgery, increasing utilization of minimally invasive approaches and allograft with associated decrease in pain and morbidity, and improvements in tools and techniques for spine surgery, many surgical procedures that were previously considered safe in the inpatient setting are now being performed in the outpatient setting [9, 30–32].

While Lad [2] analyzed 58,049 patients undergoing cervical spinal fusion for cervical disease from 1993 to 2003, which showed that the rate of complications remained stable at 10.3 % and mortality remained steady at 0.6 %. The overall complication rates of our study are typically low. The overall complication rate was about 1.71 % (29/1693). The risk ratio was 0.99, which means that compared with inpatient surgery, the risk of complication in outpatient surgery was similar. The findings are consistent with the reported literature, demonstrating no increase in complication rates or worse outcomes in patients treated as outpatient, which means that ACDF can be performed safely on an outpatient basis. As we said, due to the special location of spine, a little complication can result in serious consequences. Airway might be compromised due to cervical swelling or a hematoma and postoperative bleeding in the epidural space might lead to neurologic injury after ACDF procedure [33]. Researchers have found that all potentially life threatening complications were discovered in the first 6 h and suggested that patients could be allowed to go home after the 6-h observation period [34]. This means that the patients with outpatient surgery may not have a fatal risk compared with those with inpatient surgery, when they go back home after an observation of 6 h. Some other complications like airway swelling and respiratory compromise might peak at the second and third postoperative day [35]. Dysphagia and hematoma are the two most common postoperative complications, which should be paid attention to during or after the operation.

Besides, some studies have also reported that outpatient spine surgery has a significant cost advantages over hospital-based surgery. Anterior cervical discectomy performed in the outpatient setting may carry significant total cost savings, compared with inpatient treatment. Erickson [36] reported that the cost savings ranges from $4000 to $8000 with outpatient ACDF compared with inpatient. Cost containment may be a primary driver for performing more surgical procedures on an outpatient setting. Outpatient surgery also offers a potential benefit that may reduce the risks associated with the inpatient hospital setting, which lead to a more rapid recovery and higher patient satisfaction after the spine surgery.

Although this study suggests that outpatient instrumented ACDF is safe, there are some limitations in our study. Though the studies in our systematic review did not suggest an increased risk of complication with outpatient cervical spine surgery, the strength of evidence on the safety and value of outpatient ACDF remains scarce. In this study, the limited evidence available prevents some conclusions. But there are few studies that have been done, and some of them are of poor quality, indicating a need for further well-designed and prospective studies. Further study is needed to more clearly define the role of outpatient cervical spine surgery. Another limit is that compared with patients in outpatient surgery, inpatients had higher baseline risk factors including older, weaker and in poorer health, which implies a selection bias. It is not surprising that large number of complications were observed. Also a lead time bias may exist for more time course of care received in the inpatient setting, which allowed more detection and reporting of complications being observed.

## Conclusion

Based on the meta-analysis results, it is not associated with higher complication rates as compared with inpatient setting group and no difference of morbidity between outpatient and inpatient was found. Because of short operative time and moderate postoperative pain, we believe that outpatient cervical spine surgery is a safe and convenient alternative procedure, which also decrease the cost of care. Besides, postoperative complications including dysphagia and hematoma should be noticed.

## Abbreviations

ACDF: anterior cervical discectomy and fusion
CI: confidence interval
